# Surface Functionalised Parenteral Nanoemulsions for Active and Homotypic Targeting to Melanoma

**DOI:** 10.3390/pharmaceutics15051358

**Published:** 2023-04-28

**Authors:** Federica Foglietta, Annalisa Bozza, Chiara Ferraris, Luigi Cangemi, Valentina Bordano, Loredana Serpe, Katia Martina, Loretta Lazzarato, Stefania Pizzimenti, Margherita Grattarola, Marie Angele Cucci, Chiara Dianzani, Luigi Battaglia

**Affiliations:** 1 Dipartimento di Scienza e Tecnologia del Farmaco, Università degli Studi di Torino, 10124 Torino, Italy; 2 Dipartimento di Scienze Cliniche e Biologiche, Università degli Studi di Torino, Corso Raffaello 30, 10125 Torino, Italy; 3 Nanostructured Interfaces and Surfaces (NIS) Interdepartmental Centre, Università degli Studi di Torino, 10125 Torino, Italy

**Keywords:** nanoemulsions, targeting, proteins, melanoma

## Abstract

Despite recent progressions in cancer genomic and immunotherapies, advanced melanoma still represents a life threat, pushing to optimise new targeted nanotechnology approaches for specific drug delivery to the tumour. To this aim, owing to their biocompatibility and favourable technological features, injectable lipid nanoemulsions were functionalised with proteins owing to two alternative approaches: transferrin was chemically grafted for active targeting, while cancer cell membrane fragments wrapping was used for homotypic targeting. In both cases, protein functionalisation was successfully achieved. Targeting efficiency was preliminarily evaluated using flow cytometry internalisation studies in two-dimensional cellular models, after fluorescence labelling of formulations with 6-coumarin. The uptake of cell-membrane-fragment-wrapped nanoemulsions was higher compared to uncoated nanoemulsions. Instead, the effect of transferrin grafting was less evident in serum-enriched medium, since such ligand probably undergoes competition with the endogenous protein. Moreover, a more pronounced internalisation was achieved when a pegylated heterodimer was employed for conjugation (*p* < 0.05).

## 1. Introduction

Targeted therapies and immunotherapies are currently practiced as adjuvant approaches against melanoma specifically in several types like high-risk stage IIB/C (primary cutaneous tumour thickness > 2.0 mm) and stage III (with metastasised lymph nodes) and as advanced chemotherapy in stage IV (metastatic). Indeed, since nearly half of the human melanomas possess a V-RAF murine sarcoma viral oncogene homolog B (BRAF) mutation, BRAF and Mitogen-activated protein kinase kinase (MEK) inhibitors are used for targeted therapies, but with the relevant limitation of chemo-resistance.

Therefore, advanced melanoma still represents a life threat, pushing to optimise new nanotechnology approaches, addressed to improve specific drug delivery to the tumour, with the aim to increase therapeutic efficacy and to reduce drug doses employed, as well as unwanted side effects [1,2,3]. This can be achieved thanks to their tuneable size and surface features, which makes them capable to cross biological barriers, selectively accumulate in the tumour instead of non-target tissue [4], prevent drug chemical and/or biological degradation, and slow drug clearance from the bloodstream. In particular, they can be surface functionalized with targeting moieties, such as antibodies or ligands for specific antigens over-expressed on melanoma cells [5]. Moreover, multiple drugs can be co-loaded in combined nanomedicines, allowing the separation of incompatible compounds into physically distinct environments, to achieve synergy of action among distinct mechanisms, and to overcome chemo-resistance [6]. Within this context, hybrid nanomaterials represent a new paradigm, owing to their composite structure, drug-loading capability, and bioengineering-based approach [7,8,9].

Nonetheless, up-to-date lipid (liposomes, solid lipid nanoparticles, nanoemulsions) and polymeric (micelles, nanospheres, nanoparticles, dendrimers) nanosystems are the most frequently proposed for melanoma therapy [5]. Among nanotechnologies available, recent interest has grown up in injectable lipid nanoemulsions, since they allow foreseeing a faster translation to humans. Indeed, based upon their long history of safe clinical usage in parenteral nutrition, as well as in drug delivery aimed at pain control (anesthetics, diazepam, corticosteroids, and anti-inflammatory drugs) [10], lipid nanoemulsions have recently been proposed to deliver combined chemotherapy/immunotherapy for melanoma treatment [11,12]. Anyway, given that metastases diffused in the whole organism are the main issue in advanced melanoma, suitable targeting approaches should be adopted to target distant sites from the primary mass [13,14].

For this purpose, two different strategies can be followed, by exploiting the targeting potential of endogenous proteins. The first is active targeting, obtained by chemical grafting of a specific protein ligand whose receptor is over-expressed in melanoma. Alternatively, homotypic targeting is based upon the interaction among those adhesion proteins, which are present on the surface of cancer cells, and that can be used to coat the surface of nanoemulsion droplets [15]. Transferrin (TRF) has been frequently employed as the ligand for active targeting [16,17,18], since it shows a relatively high interspecies equivalence with respect to receptor binding [19] and its receptor is over-expressed in melanoma [16]. Literature evidence shows that nanoemulsions can be successfully conjugated to proteins by using maleimide-based linkers [20]. On the other side, promising cell membrane coating methods have been explored and utilised extensively for homotypic targeting. Therefore, cell membrane fragments (CMF) obtained from various source cells (e.g., red blood cell, immune cell, cancer cell, platelet, and fusion cell membranes) can be endowed with excellent properties, such as long blood circulation, immune escape, and targeting ability [21,22,23,24,25,26,27]. CMF are generally obtained by cell lysis in hypotonic buffer, while CMF wrapping of nanosystems can be achieved owing to several methods, including ultrasounds, homogenisation, and extrusion. Within this concern, given that injectable nanoemulsions can undergo sterile filtration, co-extrusion with CMF by means of syringe filters (220 nm) can be exploited for nanoemulsion coating.

In this experimental work, Intralipid^®^ 10% (IL) was used as the injectable nanoemulsion, to investigate the two above-mentioned targeting strategies, by means of either TRF grafting or CMF wrapping. TRF was conjugated to nanoemulsion lipid droplet through a maleimide–thiol chemistry. CMF were obtained from the B16-F10 murine melanoma cells in the perspective of further investigations in established animal models [11,12], Additionally, BRAF-mutated D4M mouse cells were separately investigated as a CMF source, which might be useful for future studies addressing chemo-resistance in BRAF-mutated mouse melanoma models. Physico-chemical characterisation of targeted IL was performed, including western blot analysis of CMF proteins used to wrap IL. Moreover, in order to assess the efficacy of the two above-mentioned targeting methods, preliminary in vitro internalisation studies were performed on B16-F10 melanoma cells, through cytofluorimetric assays with 6-coumarin (6-CUM) labelled formulations.

## 2. Experimental

### 2.1. Chemicals

1-ethyl-3-(3-dimethylaminopropyl)carbodiimide (EDC), 2-iminothiolane, 6-CUM, acetonitrile (ACN), chloroform, cysteine, dimethylsulfoxide (DMSO), Dowex 50WX4, ethanol, human TRF, N-hydroxysuccinimide (NHS), stearic acid, stearylamine, tris(2-carboxyethyl)phosphine (TCEP), tromethamol (TRIS), and Ponceau S P3504 were from Sigma-Aldrich (St. Louis, MO, USA). Hydrochloric acid (HCl), phosphotungstic acid, Millipore Amicon^®^ Centriflo CF50 tubes, and Millipore MilliQ system (for deionised water) were from Merck (Darmstadt, Germany). Ethylendiaminotetraacetic acid (EDTA), hexa-hydrate magnesium chloride (MgCl_2_), potassium chloride (KCl), and sodium chloride (NaCl) were from Carlo Erba (Cornaredo, Italy). 3-Maleimidobenzoic acid N-hydroxysuccinimide ester (MBS), Agarose^®^ CL 4B, anhydrous magnesium sulphate (MgSO_4_), isopropanol (IPA), and trifluoroacetic acid (TFA) were from Alfa Aesar (Haverhill, MA, USA). Di-amino Polyethylen Glycol 2000 MW (diamino-PEG) was from Iris Biotech (Marktredwitz, Germany). IL was from BBraun. Sephadex^®^ G25 was from GE Healthcare (Chicago, IL, USA). 220 nm sterile filters were from GVS (Bologna, Italy). β-actin monoclonal antibody (sc-47778) and pan-cadherin monoclonal antibody (sc-59876) were from Santa Cruz Biotechnology, Inc. (Heidelberg, Germany). CD324 (E-Cadherin) Monoclonal Antibody (DECMA)—Alexa Fluor™ 488, was from eBioscience, Thermo Fisher Scientific (Milano, Italy). All other chemicals were of analytical grade and used without any further purification.

### 2.2. Cells

B16-F10 murine melanoma cells were purchased from the American Type Culture Collection (ATCC; Manassas, VA, USA). D4M cells, a mouse melanoma-engineered cell line harbouring the BRAFV600E mutation, were a gift from D.W. Mullins, Department of Medicine, Norris Cotton Cancer Center, Geisel School of Medicine at Dartmouth, Lebanon, NH, USA [28]. B16-F10 cells were cultured in RPMI 1640 medium (Sigma-Aldrich, St. Louis, MO, USA), while D4M was in Dulbecco’s Modified Eagle Medium (DMEM—Sigma-Aldrich, St. Louis, MO, USA). All culture media were supplemented with 10% Fetal Calf Serum (FCS; PAA Laboratories, Pasching, Austria), streptomycin (100 µg/mL) and penicillin (100 units/mL), and L-glutamine (2 mmol/L) (both from Sigma-Aldrich, St. Louis, MO, USA). Cell lines were cultured in a 5% CO_2_, 37 °C incubator.

### 2.3. Methods

#### 2.3.1. Linkers Synthesis and Characterisation

Two different maleimide-linkers, N-Octadecil-3-Maleimido-Benzamide (ST-MBS), and stearyl-PEG-maleimide heterodimer (ST-PEG-MBS) were synthesised and characterised owing to previously established methods [29] (See Supplementary Materials and Figure S1).

#### 2.3.2. IL Functionalisation with TRF

First, thiolated TRF was synthesised with a 1:2 TRF/2-iminothiolane molar ratio [29,30]. To this aim, three stock solutions were prepared separately in distilled water: (1) 285 μg/mL TCEP (a mild reducing agent used to prevent thiol groups dimerisation to disulfide bonds); (2) 70 μg/mL 2-iminothiolane; (3) 1 mg/mL TRF. The reaction was carried out as follows: 1 mL TRF (0.0025 μmoles), 50 μL TCEP (0.011 μmoles), and 50 μL 2-iminothiolane (0.005 μmoles) stock solutions were mixed with 300 μL 0.01 M phosphate buffer pH 7.40, and kept under magnetic stirring for 1 h, followed by size exclusion (Sephadex^®^ G25) purification.

IL surface grafting was achieved by dissolving either 1 mg (0.41 μmoles) ST-PEG-MBS or 0.2 mg ST-MBS (0.43 μmoles) in 1 mL of IL; in the case of ST-MBS, 1 mg linker was pre-dissolved in 50 μL of DMSO and subsequently 10 μL of this solution was added to 1 mL of IL. Then, thiolated TRF was added to the IL-linker mixture in variable amounts: 0.2 and 1 mg (0.0025 and 0.0125 μmoles), respectively. After overnight reaction under magnetic stirring, the excess of maleimide was saturated in 4 h with 100 μL of a 1.6 mg/mL cysteine solution (0.16 mg–1.32 μmoles). Finally, TRF grafted IL was purified from the reaction mixture either by ultracentrifugation at 26,000 rpm for 15 min (Allegra^®^ 64R centrifuge, Beckman Coulter, Palo Alto, CA, USA), followed by re-suspension in 1 mL deionised water, or by size exclusion (Agarose^®^ CL 4B), followed by concentration to 2 mL with Amicon^®^ Centriflo CF50 tubes (2000 rpm for 15 min—Rotofix 32 centrifuge, Hettich, Tuttingen, Germany).

#### 2.3.3. IL Wrapping with CMF

CMF were isolated from B16-F10 and D4M murine melanoma cells, working under a sterile hood and in an ice bath [31]. Approximately 10^6^ cells were detached from the culture medium with sterile 0.58 mg/mL EDTA. The pellet was re-suspended with a micropipette in 1 mL of 0.75 mg/mL KCl, 0.41 mg/mL MgCl_2_ in 0.02 M TRIS-HCl sterile hypotonic buffer, and it underwent lysis in a glass-homogeniser (20 strokes), followed by centrifugation at 3200× *g* for 5 min (Allegra^®^ 64R centrifuge, Beckman Coulter, Palo Alto, CA, USA), in order to extract CMF in the supernatant. This procedure was repeated twice, then CMF were isolated from the supernatant by centrifugation at 19,800× *g* for 20 min (Allegra^®^ 64R centrifuge, Beckman Coulter, Palo Alto, CA, USA), and CMF suspension was obtained from the pellet with a micropipette in 1 mL of sterile normal saline.

IL wrapping by B16-F10/D4M CMF was achieved by co-extrusion of 200 μL CMF suspension and 1 mL IL with 220 nm sterile filters.

#### 2.3.4. Characterisation of Nanoemulsions

##### Particle Size, Shape, Zeta Potential

The dynamic light scattering technique (DLS; 90 Plus, Brookhaven, NY, USA) was used to determine the mean droplet size, polydispersity index (PDI), and Zeta potential of the IL-based formulations, at 25 °C, and in triplicate. Measurement angles were 90° for particle size and 15° for Zeta potential. Optical microscopy was performed by a DM2500 microscope (Leica Microsystems, Wetzlar, Germany), equipped with a Moticam 480 camera (Motic, Barcelona, Spain). Transmission Electronic Microscopy (TEM, High Resolution JEOL 300 kV) was performed via IL-negative staining with 1% phosphotungstic acid [11].

##### Protein Analysis

Proteins grafted to the IL surface (μg/g lipid) and in the IL supernatant (μg/mL) were quantified by high-pressure liquid chromatography (HPLC), after nanoemulsion ultracentrifugation and protein extraction.

For TRF functionalised IL, the procedure was as follows: 250 μL of the purified formulations were centrifuged at 62,000× *g* (Allegra^®^ 64R centrifuge, Beckman Coulter, Palo Alto, CA, USA) for 5 min; the supernatant was injected in HPLC as is, while the lipid pellet was re-suspended in 50 μL of a protein extraction solution (46% water 42% IPA, 12% ACN, 0.1% TFA) and the mixture centrifuged again at 62,000× *g* for 5 min, before HPLC injection.

For CMF-wrapped IL since 200 μL B16-F10/D4M CMF suspensions were used for each mL of IL, preliminarily, the total protein content of CMF suspensions was determined: CMF were separated from the supernatant by centrifugation at 18,000× *g* for 5 min; the supernatant was injected as is, while the pellet was extracted with 50 μL of protein extraction solution, prior to injection in HPLC.

Then the proteins grafted to the IL droplet of B16-F10/D4M CMF-wrapped IL were determined as follows: 6 mL of the formulations obtained were centrifuged at 62,000× *g* for 10 min. The supernatant was injected as is, while the lipid pellet was re-suspended in 5 mL of the above-mentioned protein extraction solution and centrifuged again at 62,000× *g* for 20 min; the obtained supernatant was transferred to a glass test tube and dried under nitrogen flow; the residue underwent two further re-suspension/centrifugation/drying cycles with 1 mL an 200 μL of protein extraction solution, which was finally injected into HPLC.

Qualitative determination of adhesion proteins in B16-F10/D4M CMF was performed by Western Blot (WB) and immuno-fluorescence. Through WB analysis 2 model protein markers were identified in cell lysates and CMF suspensions: pan-cadherins (membrane protein model) and β-actin (cytoplasmatic protein model). Before hybridisation, total protein amount in the samples was estimated by Bradford assay and Sodium Dodecyl Sulphate—PolyAcrylamide Gel Electrophoresis (SDS-PAGE) with Ponceau S staining, in order to analyze equal protein concentrations in WB. To this aim, CMF suspensions were concentrated from 1 mL to 50 μL. Protein bands were quantified by densitometry (Image J, Bristol, UK—version 1.53i). In immunofluorescence, either 200,000 B16-F10 cells (suspended in 0.2 mL of PBS), or 100 μL of B16-F10 CMF suspension, or 100 μL of B16-F10 CMF-wrapped IL were incubated for 1 h at room temperature with 0.5 μg/mL anti-E-cadherin antibody, then centrifuged for 10 min (96× *g* for cells, 18,000× *g* for CMF and 62,000× *g* for IL), and re-suspended in 0.2 mL PBS. Afterward, a small drop of sample from the suspension was put on glass for fluorescence and observed by microscopy (DM2500, Leica Microsystems, Wetzlar, Germany, equipped with a Moticam 480 camera, Motic, Barcelona, Spain).

#### 2.3.5. HPLC

RP HPLC analysis for TRF and CMF proteins was carried out at 75 °C, using a 300 Å pore C8 column (Tecnokroma Tracer Excel, Barcelona, Spain 25 × 0.4 cm). The gradient was performed between eluent A (0.1% TFA) and eluent B (70% IPA, 20% ACN, 10% water, 0.1% TFA): 0 min: 90% A; 15 min: 40% A; 20 min: 40% A; 23 min 90% A. The flow rate was maintained at 1 mL/min. The UV detector was set at λ = 220 nm. The retention time was 10.5 min for TRF and 12.5 min for CMF proteins.

#### 2.3.6. Cellular Uptake of Fluorescently Labelled Nanoemulsions

In order to perform uptake studies in B16-F10 melanoma cells, IL, either blank or surface modified i.e., IL ST-MBS TRF, IL ST-PEG-MBS TRF, and IL B-16-F10 CMF were labelled with 0.1 mg/mL 6-coumarin (6-CUM) fluorescent probe [11,20,32,33,34,35]. To this aim 50 μL of a 2 mg/mL stock solution of 6-CUM in DMSO were added to 1 mL of nanoemulsion, followed by mixing with a micropipette.

Internalisation was investigated by incubating the formulations under study with B16-F10 cells for 1 h at 37 °C within an incubator with 5% CO_2_. The internalisation was evaluated in an FCS-enriched culture medium, but, in the case of TRF-functionalised IL, also FCS-free medium was used. In the former case, the incubation was carried out on cells suspended in tubes, while in the latter one on adherent cells, plated on 6-well plates 14 h before the assay. 200,000 cells were used per condition, and the correct ratio between cells and labelled formulations was preliminarily established, to avoid saturation of cell uptake. Then, cells were centrifuged at 96× *g* (NF 200, Nuve, Ankara, Turkey) for 10 min, re-suspended in 0.5 mL phosphate-buffered saline (PBS), and analyzed with an Accuri C6 (BD Biosciences, Milan Italy) flow cytometer (considering 10,000 events and medium flow rate). Any cell debris with low forward light scatter (FSC) and side light scatter (SSC) were excluded from the analyses. Untreated B16-F10 cells were used as controls (Ctrl). 6-CUM fluorescence was expressed as the integrated mean fluorescence intensity (iMFI), that is, fluorescence-positive cells frequency per median fluorescence intensity.

### 2.4. Statistical Analysis

Data were expressed as mean ± standard mean error (SEM). Statistical analyses were performed using a one-way ANOVA and post-ANOVA Tukey’s test (Prism Graphpad 5.0, Graphpad Software, San Diego, CA, USA) in order to compare surface functionalised vs. blank IL.

## 3. Results

### 3.1. Physico-Chemical Characterisation of Nanoemulsions

The yield of chemical grafting with TRF depends on the protein amount used for the reaction, on the linker, and the purification method employed. Using a higher TRF amount for reaction results in a proportionally higher amount of protein grafted. Moreover, ST-MBS is more lipophilic than ST-PEG-MBS, being more strictly associated with the lipid matrix: this causes a stronger anchoring of the hydrophilic TRF to the lipid droplets, with respect to that in the supernatant. Finally, the purification from the reaction mixture is very important. Indeed, ultracentrifugation allows a sharp phase separation, whereas hydrophilic un-reacted compounds can be easily retained in the supernatant and eliminated. However, re-suspension could lead to a severe alteration at the lipid/water interface, causing protein leakage toward the external phase. This is more evident for hydrophilic ST-PEG-MBS. Size exclusion, instead, allows improved retention of conjugated TRF, even if a concentration process is needed to recover the original volume. In B16-F10 and D4M CMF suspensions, all the proteins are recovered in the CMF pellet after centrifugation. Nonetheless, after extrusion with IL, there is a partial protein leakage towards the external phase.

However, none of the procedures employed caused a significant particle size increase, compared to blank IL. Of note, the PDI of CMF is very high, much more than nanoemulsions. This means that several size populations are present, including nano and micro-vesicles (Figure S2). Zeta potential, instead, is affected by the conjugated proteins. Blank IL is associated to a sharp negative charge, due to the lecithin emulsifier. TRF, which is endowed to a mean positive charge, leads to a general decreasing trend in the absolute Zeta potential value of conjugated formulations, which, however, is significant only when 1 mg TRF is used for reaction, ST-MBS as the linker, and ultracentrifugation/re-suspension for purification: probably such conditions allow the grafting of a major amount of TRF nearby the interface of IL droplets. B16-F10 are associated with a significantly less negative Zeta potential compared to blank IL, while D4M ghost to a more negative one. A consequent trend indicates a shift towards intermediate Zeta potentials for ghost-wrapped formulations, with respect to ghost suspensions and blank IL.

In Table 1, the physico-chemical characterisation of surface functionalised IL is reported.

#### Surface Coating Characterisation of CMF-Wrapped IL

**Figure 1:**
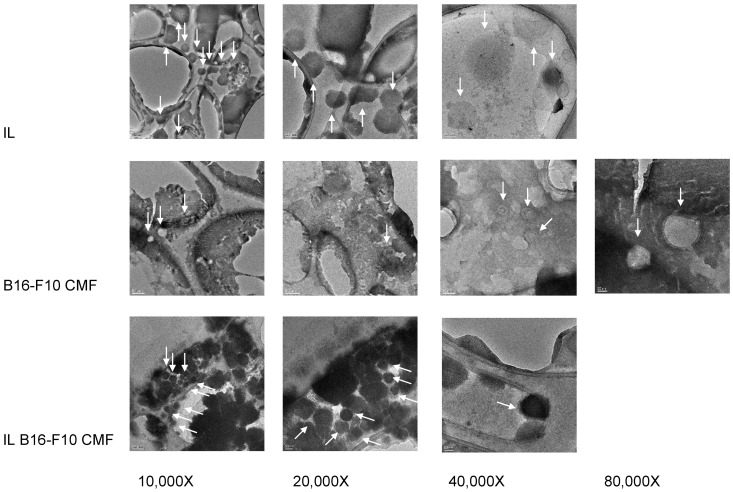
Transmission electronic microscopy (TEM) of Intralipid^®^ 10% (IL), B16-F10 cell membrane fragments (CMF), and CMF-wrapped IL (IL CMF B16-F10). Arrows indicate either the IL droplet or the CMF. Scale bars: 10,000× 200 nm; 20,000× 100 nm; 40,000× 50 nm; 80,000× 20 nm.

The surface coating of CMF-wrapped IL was further characterised. Indeed, CMF wrapping process was followed step-by-step by optical microscopy (Figure S2). Moreover, TEM images were obtained for B16-F10 CMF and CMF-wrapped IL, and blank IL (Figure 1).

As it can be noted, CMF are vesicle-like structures; compared to blank nanoemulsion, wrapped IL results in a different contrast in TEM images, whereas a surface layer surrounds the lipid droplet. Such a coating could be due to cell membrane components, both phospholipids and proteins.

**Figure 2:**
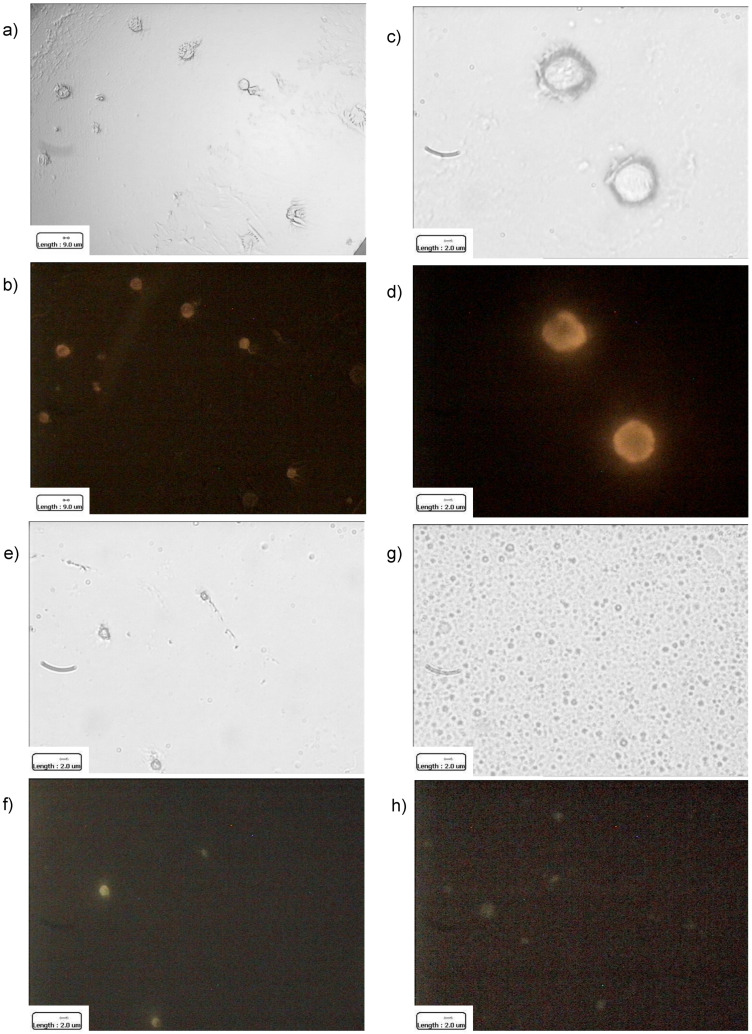
Optical microscopy. 100× magnification: B16-F10 cells (**a**): normal light; (**b**): E-cadherin immuno-fluorescence). Scale bar: 9 μm. 630× magnification: B16-F10 cells (**c**): normal light; (**d**): E-cadherin immuno-fluorescence), B16-F10 cell membrane fragments (CMF) (**e**): normal light; (**f**): E-cadherin immuno-fluorescence), B16-F10 CMF-wrapped Intralipid^®^ 10% (IL B16-F10 CMF) (**g**): normal light; (**h**): E-cadherin immuno-fluorescence). Scale bar: 2 μm.

Therefore, E-cadherin was taken as a protein model, in order to qualitatively track protein presence on CMF and CMF-wrapped IL, throughout all the coating steps. Given the variable expression of E-cadherin on melanoma cells [36,37,38,39], flow cytometry was preliminarily performed to assess E-cadherin antibody binding to B16-F10 cells. E-cadherin antibody showed a good specificity for cells, even with low iMFI (Figure S3), accounting for a weak E-cadherin expression, as identified also by Lorena Lobos-González [40]. However, despite such concern, which resulted in a low signal in fluorescence microscopy, the presence of E-cadherin was appreciated in B16-F10 cells, CMF and CMF-wrapped IL (Figure 2).

**Figure 3:**
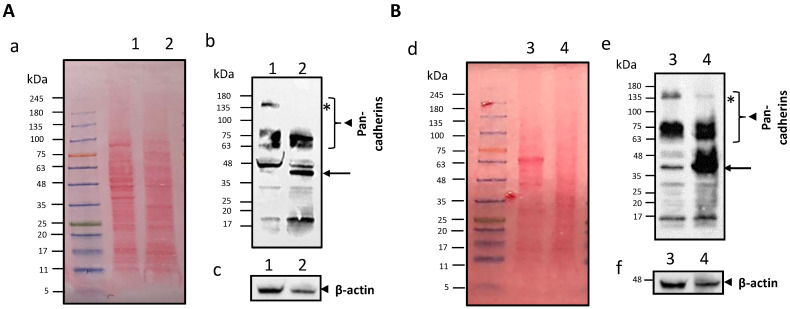
Cell membrane fragment (CMF) protein characterisation in B16-F10 (panel (**A**)) e D4M (panel (**B**)) melanoma cells. Panel (**A**): (**a**) Sodium Dodecyl Sulphate–PolyAcrylamide Gel Electrophoresis (SDS-PAGE) protein analysis of B16-F10 cancer cell lysate (lane 1), and B16-F10 CMF (lane 2). Samples were run at equal protein concentrations and stained with Ponceau S, showed by colored picture. (**b**,**c**) WB analysis for membrane-specific and intracellular protein markers. Samples were run at equal protein concentrations and immunostained against the membrane marker pan-cadherin (**b**), and the cytosolic marker β-actin (**c**), showed with black and white picture by chemiluminescent signal. Panel (**B**): (**d**) SDS-PAGE protein analysis of D4M cancer cell lysate (lane 3), and D4M CMF (lane 4). Samples were run at equal protein concentrations and stained with Ponceau S, showed by colored picture. (**e**,**f**) WB analysis for membrane-specific and intracellular protein markers. Samples were run at equal protein concentrations and immunostained against the membrane marker pan-cadherin (**e**) and the cytosolic marker β-actin (**f**), showed by black and white pictures by chemiluminescent signal. Arrows in (**b**,**e**) indicate the ~38 kDa size band as a possible plasma membrane-embedded fragment of E-cadherin. * in (**b**,**e**) indicate the cadherin precursors, i.e., the heaviest band > 135 kDa marked.

However, compared to parent cells, CMF should be enriched in cell membrane proteins, that is, those responsible for homotypic targeting compared to cytoplasmatic ones. To this aim, two types of highly expressed proteins were considered as models for WB analysis of B16-F10 cells and CMF: cadherins as a model of membrane proteins, and β-actin as a cytoplasmic one [31]. D4M cells and CMF were also investigated for comparison purposes (Figure 3).

The monoclonal anti-pan cadherin antibody used in this study can react extensively with all known members of the cadherin family, such as E-, N- or P-cadherins, since it can recognise the cytoplasmic C-terminal region, the most conserved region of the cadherins [41]. According to the manufacturer’s indications, the molecular weight of cadherins ranges between 60 and 120 kDa. This broad range of molecular weight is not only due to the different forms of cadherins detected by the antibody but also because every single class of cadherins can undergo several post-translational or post-transcriptional modifications, which can modify their molecular weight. Indeed, glycosylation [42], alternative splicing [43], or fragmentation via proteolytic cleavage [44] can be responsible for such extreme size variability. In melanoma cells, a truncated form of P-cadherin with 50 kDa weight was identified [45], as well as an 80kDa weight E-cadherin alternative splicing variant [43]. Moreover, a 38 kDa intracellular C-terminal fragment of E-cadherin (E-cad/CTF1), embedded within the plasma membrane, can be formed after the cleavage of the mature form in several cell types [44]. Additionally, the anti-pan cadherin antibody can also detect precursor cadherin molecules, with a molecular weight ≥130 kDa [46,47]. Following the synthesis of the precursors, the maturation in the Golgi network involves the proteolytic cleavage near the N-terminus at a specific recognition sequence and glycosylation. Precursors of the cadherin molecules are detected in the cytosol but they are not expressed in the plasma membrane [46,48].

As shown in Figure 3, in CMF with respect to the cell lysate we didn’t observe any difference in the expression of 60–120 kDa proteins, corresponding to the cadherin mature forms in both cell lines. However, there was a significative inhibition of the expression of the cadherin precursors, the heaviest band > 135 kDa marked with an asterisk, and an increase of the ~38 kDa size band indicated with an arrow, a possible plasma membrane-embedded fragment of E-cadherin. A significative decrease of the cytoplasmic marker β-actin was found in CMF with respect to the cell lysate in both cell lines. The densitometry analysis is reported in Figure S4.

### 3.2. Cytofluorimetric Uptake Studies of Lipid Nanoemulsion in B16-F10 Cells

Uptake studies of the considered lipid nanomemulsion were performed on B16-F10 cells, by using 6-CUM as an established fluorescent probe to label the lipid matrix of IL (Figure S5) [49], whose cell internalisation may undergo a quick saturation [11]. First, preliminary investigations were performed in order to optimise IL/cell number and 5 experimental conditions were considered (Figure S6). Given the clear difference in iMFI between condition 4/5 compared to 1/2/3, with functionalised formulations, the markedly low ratio of 1 µL IL vs. 5 × 10^6^ cells were maintained, because it may work below cell saturation, allowing us to appreciate the effect of functionalisation. Moreover, any unexpected toxicity was detected in such conditions.

**Figure 4:**
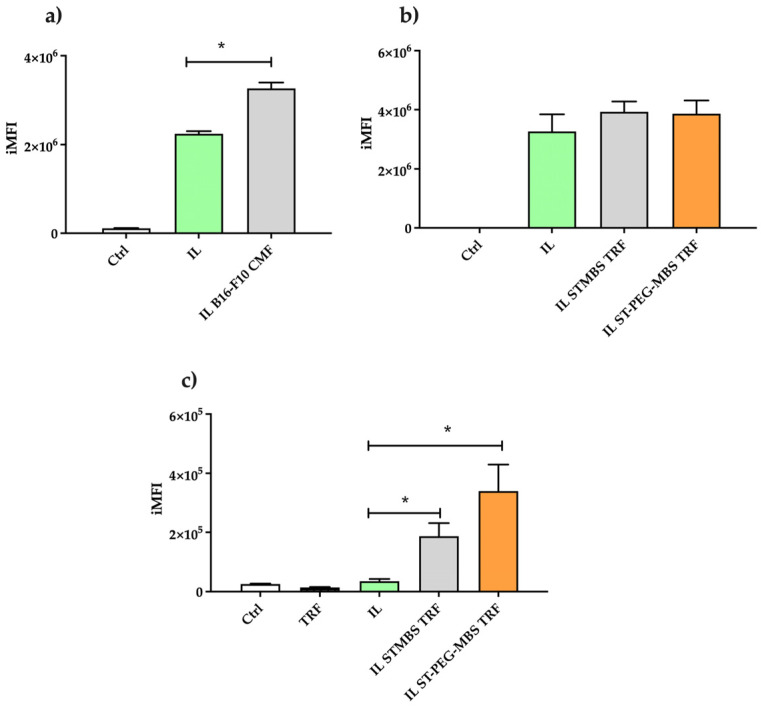
Internalisation of fluorescently labelled Intralipid^®^ 10% (IL), blank and surface functionalised, in B16-F10 cells by flow cytometry. Influence of surface functionalisation with B16-F10 cell membrane fragments (CMF) (**a**) and transferrin (TRF) (**b**) in Fetal Calf Serum (FCS)-enriched medium, and of TRF functionalisation in FCS-free medium (**c**). TRF (1 mg) was grafted of IL droplet surface by means of either N-Octadecil-3-Maleimido-Benzamide (ST-MBS) or Stearyl-PEG-maleimide heterodimer (ST-PEG-MBS). Statistical significance versus untreated IL: * *p* ≤ 0.05.

Noteworthy, since TFR grafted IL was optimised with 1 mg protein used for reaction and size exclusion purification, such conditions were selected for the internalisation study: ST-MBS and ST-PEG-MBS were alternatively used for TRF conjugation, aiming for comparison purposes. On the other side, B16-F10 CMF-wrapped IL was used, owing to the homotypic targeting with the corresponding cells (Figure 4).

As it can be noticed, the uptake of 6-CUM in CMF wrapping was higher compared to IL alone, allowing us to hypothesise an enhanced cell internalisation of IL. Instead, the effect of TRF grafting was less evident in an FCS-enriched medium, probably because plasma-derived FCS contains TRF, which may compete for membrane receptor binding with the IL-grafted ligand [50]. However, TRF functionalisation exerts its effect in the FCS-free medium, even if in this case a lower iMFI was detected for all the samples under study. Noteworthy, a more pronounced internalisation was achieved when ST-PEG-MBS is employed (*p* < 0.05), probably due to the PEG spacer, which allows an improved ligand binding to the receptor.

## 4. Discussion

### 4.1. Physico-Chemical Standpoint

Compared to alternative nanocarriers, injectable lipid nanoemulsions offer several advantages in terms of biocompatibility, physico-chemical stability, easy sterilisation (by steam and/or filtration), and high scalability for large batch production [51]. In particular, their physico-chemical stability allows them to freely perform chemical reactions at the surface of droplets, without altering their size; on the other side, sterile filterability can be exploited for CMF wrapping by extrusion, achieving simultaneously a sterile and CMF-coated nanoemulsion. Of note, as evidenced by experimental results, surface functionalisation, either chemical or biological, did not affect nanoemulsion size.

Maleimide-thiol chemistry was selected for TRF grafting for its chemical selectivity [52]. Indeed, the free thiol group, chemically inserted on the TRF molecule, is absent from the lipid matrix, as well as from most of the potential drug candidates that could be loaded, while the maleimide moiety reacts selectively with it. The main chemical concern of such an approach is given by oxidation to disulfide, which can be easily managed by working in a mild reducing environment [29,30]. Furthermore, such interface reaction occurs with an excess of maleimide linker with respect to TRF, therefore saturation of excess maleimide with free thiols moieties (such as cysteine) is needed, in order to avoid unwanted chemical binding of functionalised IL to biological molecules exhibiting a free thiol group (i.e., albumin) [53]. Noteworthy, chemical grafting is characterised by a low yield, being an interface reaction. Moreover, as a hydrophilic protein, conjugated TRF tends to migrate in the external phase of the purified nanoemulsion, especially when the pegylated linker is used for grafting. Nonetheless, literature evidence assesses that the PEG spacer accounts for improved interaction of the ligand with its receptor [54]. Purification by size exclusion also allows better recovery of the grafted protein, probably because it avoids the sharp phase separation occurring during the ultracentrifugation process, and therefore it has been selected for the following internalisation studies.

Proteins, instead, can be completely isolated from CMF after centrifugation, and, therefore, it can be supposed that they are associated with the vesicles. Noteworthy, western blot analysis allowed us to appreciate an increase of cell membrane proteins in CMF, compared to cytoplasmatic ones. However, the co-extrusion of CMF with IL causes a certain protein leakage towards the external phase, even if this should not be detrimental to the targeting properties, as assessed by the presence of E-cadherin on the surface of CMF-wrapped IL.

### 4.2. Biological Standpoint

One of the fascinating advantages of nanocarriers in cancer therapy is the possibility of surface modification, which allows the delivery of the cargo specifically to the target cells, avoiding off-target delivery and associated side effects [55]. TRF receptors are often over-expressed on the surface of a wide variety of solid tumors including melanoma [56,57]. Therefore, TRF grafting onto the nanoemulsion surface could improve their systemic delivery to the cancer site. TRF-decorated nanoparticles were successfully employed to target melanoma in an in vivo model of B16-F10 melanoma tumour-bearing mice [17,58]. On the other side, among the different strategies developed in the latest few years for nanotherapeutics targeted delivery, homotypic membrane-membrane recognition is one of the most promising and efficient [59]. Indeed, cell membrane coatings, which provide a streamlined method for creating multifunctional and multi-antigenic drug delivery systems, have increasingly attracted the attention of researchers, as they can boost biocompatibility, immune evasion, tumour targeting, and treatment efficiency [60]. In particular, tumour cell-membrane-coated vesicles and nanoparticles, obtained by melanoma B16-F10 cells, showed promising targeting and therapeutic efficacy in vitro and in vivo melanoma models [31,61]. This could be ascribed to the selective affinity for melanoma of the complex protein coating, that CMF retains from the parent cells [62,63]. For this purpose, in our work, we demonstrated a membrane protein enrichment in B16-F10 CMF, with respect to corresponding cells. Indeed, we demonstrated a lower content of the cadherin precursors and beta-actin, both not expressed in the plasma membrane. Moreover, we can suggest an enrichment of the ~38 kDa plasma membrane-embedded fragment of E-cadherin. Of note, recently TRF grafting and cell membrane coating can be merged in order to functionalise the same nanosystem. Indeed, Bidkar and colleagues demonstrated that the preparation of TRF receptor-targeted red blood cells membrane-coated nanoparticles achieved chemotherapeutic and photodynamic effects on HeLa and MCF-7 cells both growth as a monolayer and as a spheroid [64].

Despite such targeting approaches being sensitive to many variables in vivo, internalisation studies in 2D cell models can be predictive of their efficiency [61], providing that saturation of the process should be avoided, in order to obtain reliable data. Of note, in standard culture conditions, i.e., in the presence of FCS, CMF wrapping was more efficient than TRF grafting in enhancing cellular uptake. Furthermore, the removal of FCS allowed us to appreciate TRF-mediated targeting, with a more pronounced effect in the presence of the PEG spacer, as expected (Figure 4). Since it has been reported in the literature that the FCS-supplemented media formulations can influence the cultured cell physiology, extracellular vesicle production/release, and its contaminating presence of vesicular and non-vesicular particles [65], we might hypothesise that plasma TRF presence can have a role when present in FCS [50]. However, Rashid and colleagues reported that serum-devoid medium influences specific features of cancer cells, including viability, morphology, and protein expression profile (proteasome subunit alpha Type 2, chloride intracellular channel protein 1, and heat shock 70 kDa protein 5) [66], with consequent cell stress. These phenomena pose serious concerns about the supposed efficacy in vivo of the TRF approach, mainly due to the competition with the endogenous protein. The latter concern probably could be addressed by substituting the parent ligand with synthetic TRF analogues, characterised by a higher affinity for its receptor [67].

## 5. Conclusions

In this experimental work, injectable nanoemulsions were functionalised with proteins, either by means of chemical grafting with TRF for active targeting or by cancer cell CMF wrapping for homotypic targeting. This was reached thanks to their good physico-chemical stability and sterile filterability, allowing interface reactions occurrence and wrapping by extrusion, respectively, without alteration of the mean droplet size. Therefore, such functionalisation processes might be translated to nanoemulsions loaded with drugs or drug combinations for melanoma therapy.

The targeting efficiency of fluorescently labelled formulations was assessed through in vitro uptake studies in 2D melanoma cells. Despite both targeting approaches being suitable to increase internalisation with respect to unfunctionalised nanoemulsions, stronger evidence came for CMF-wrapped formulations, since TRF targeting efficiency might be limited by competition with the endogenous protein.

## Figures and Tables

**Table 1:** Nanoemulsions physico-chemical characterisation. Abbreviations: CMF: cell membrane fragments; IL: Intralipid^®^ 10%; PDI: polydispersity index; ST-PEG-MBS: stearyl-PEG-maleimide heterodimer; SE: size exclusion; ST-MBS: N-Octadecil-3-Maleimido-Benzamide; TRF: transferrin; UR: ultracentrifugation/re-suspension. Statistical analysis: * *p* < 0.05, ** *p* < 0.01 vs. blank IL.

	Linker	Purification	Mean Size (nm)	PDI	Zeta Potential (mV)	Proteins in Functionalized IL			
						μg Used in 1 mL		Recovered	
						TRF	CMF	Lipid (μg/g)	Supernatant (μg/mL)
Blank IL	-	-	290.0 ± 1.9	0.005	−39.53 ± 2.07	-	-	-	-
IL TRF	ST-MBS	UR	263.5 ± 1.3	0.185	−40.27 ± 3.77	200	-	40 ± 3.5	6.3 ± 0.4
	ST-PEG-MBS	UR	262.8 ± 3.6	0.192	−35.81 ± 1.81	200	-	16 ± 1.4	14.7 ± 1.2
	ST-MBS	UR	247.4 ± 3.0	0.022	−24.11 ± 4.31 *	1000	-	74 ± 8.7	70.7 ± 2.3
	ST-PEG-MBS	UR	252.3 ± 2.7	0.137	−35.35 ± 1.80	1000	-	60 ± 7.1	15.5 ± 1.6
	ST-MBS	SE	251.1 ± 2.0	0.131	−30.06 ± 4.55	1000	-	125 ± 11.8	26.7 ± 3.0
	ST-PEG-MBS	SE	256.1 ± 1.7	0.159	−40.33 ± 3.85	1000	-	265 ± 24.5	48.5 ± 4.5
B16-F10 CMF	-	-	642.5 ± 30.7	0.521	−18.48 ± 3.17 **	-	-	-	-
IL B16-F10 CMF	-	UR	235.7 ± 2.8	0.072	−32.81 ± 3.92	-	5.8	20.7 ± 1.7	2.5 ± 0.2
D4M CMF	-	-	748.3 ± 31.1	0.391	−49.03 ± 4.40 *	-	-	-	-
IL D4M CMF	-	UR	220.1 ± 3.2	0.180	−42.83 ± 2.70	-	7.4	36.2 ± 16.9	2.8 ± 0.8

## Data Availability

The data presented in this study are available on request from the corresponding author.

## Supplementary Materials

The following supporting information can be downloaded at: https://www.mdpi.com/article/10.3390/pharmaceutics15051358/s1, Figure S1. ^1^H-NMR spectrum of ST-PEG-MBS. Figure S2. Optical microscopy of B16-F10 and D4M cell membrane fragments (CMF) wrapping of Intralipid^®^ 10% (IL). 630× magnification. Scale bar: 2 μm. Figure S3. Flow cytometry of B16-F10 cells stained by immunofluorescence with E-cadherin antibody. Figure S4. Densitometric analysis of B16-F10/D4M cell lysate and the corresponding CMF WB showed in Figure 3. We quantified the mature forms of cadherins (60–120 kDa), the cadherin precursors (>130 kDa), the ~38 kDa size band, a possible plasma membrane embedded fragment of E-cadherin, and the cytosolic marker β-actin. Data were normalized with the densitometry measurement mad over the entire line between 5 and 245 kDa. Values are indicated in percentage of control values as the mean ± SD of three independent experiments. ** *p* ≤ 0.01 vs. C. Figure S5. Fluorescence microscopy of 6-CUM fluorescently labelled Intralipid^®^ 10% (IL). 630X magnification. Figure S6. Flow cytometry evaluation of internalization of fluorescently labelled Intralipid^®^ 10% (IL) in B16-F10 cells with different condition set: (a) fluorescence of 6-CUM over cell number; (b) mean integrated fluorescence intensity (iMFI). Conditions: (1) 1 µL IL vs. 1 × 10^5^ cells (2) 1 µL IL vs. 5 × 10^5^ cells (3) 1 µL IL vs. 1 × 10^6^ cells (4) 1 µL IL vs. 5 × 10^6^ cells (5) 1 µL IL vs. 1 × 10^7^ cells.
