# Supplementary material for: Surface Functionalised Parenteral Nanoemulsions for Active and Homotypic Targeting to Melanoma
Source: Pharmaceutics. 2023 Apr 28;15(5):1358. doi: 10.3390/pharmaceutics15051358 (PMC10222029; doi:10.3390/pharmaceutics15051358)
Supplement: Supplementary file 1 [file pharmaceutics-15-01358-s001.zip › pharmaceutics-2272542-supplementary.pdf]

Supplementary materials

ST-MBS synthesis and characterization p.s2

Stearyl-PEG-maleimide heterodimer (ST-PEG-MBS) synthesis and characterization p.s3

Figure s2 p.s4

Figure s3 p.s5

Western blot densitometry p.s6

Figure s5 p.s7

Optimization of ration between B16-F10 cells and IL p.s8

ST-MBS synthesis and characterization

4.5 mg stearylamine and 5 mg of 3-maleimidobenzoic acid N-hydroxysuccinimide ester (MBS) were dissolved in 1 mL of CHCl3 and 15 µL di triethylamine and kept reacting at 40 °C for 4 h. The chloroform phase was extracted twice with 1% NaCl and twice with distilled water to remove water-soluble byproducts. $MgSO_4$ was added to the chloroform phase and filtered. The mixture was dried from chloroform using a rotor-evaporator.

The reaction was assessed through Thin Layer Chromatography (TLC — acetic acid/chloroform/methanol 1/89/10). The product was characterized through HPLC. Analyses were performed with a YL9100 HPLC system equipped with a YL9110 quaternary pump, a YL9101 vacuum degasser and a YL9160 PDA detector, linked to YL-Clarity software for data analysis (Young Lin, Hogyedong, Anyang, Korea). Column was a Teknocroma (Barcelona, Spain) C18 mediterranea Sea 25 × 0.46 cm, photo diode array (PDA) wavelengths were set at 220–290 nm, eluent flow was 1 mL/min, gradient was 0 min: 100% water, 20 min: 100% acetonitrile, 30 min: 100% acetonitrile, 35 min: 100% water. ^{1}H and NMR spectra were recorded on a Jeol (Akishima, Japan) ECZ-R 600, at 600 MHz, using $SiMe_4$ as internal standard. The following abbreviations are used to designate peak multiplicity: s = singlet, d = doublet, t = triplet, m = multiplet, bs = broad singlet. ESI-MS spectra were recorded on a Micromass Quattro API micro (Waters Corporation, Milford, MA, USA) mass spectrometer. Data were processed using a MassLynx System (Waters).

TLC: ST-MBS R_f 0.88, MBS R_f 0.75, stearylamine R_f 0.28.

^{1}H NMR (600 MHz, CDCl3) δ 7.76-7.74 (m, 1H), 7.73-7.71 (m, 1H), 7.54-7.52 (m, 1H), 7.49-7.47 (m, 1H), 6.87 (s, 2H), 6.19-6.15 (m, 1H), 3.45-3.40 (m, 2H), 1.26-1.22 (m, 32H), 0.86 (t, J = 6 Hz, 3H).

ESI-MS $[M+H]^+$: m/z 468.

HPLC PDA: R_t = 25.6 min.

ST-PEG-MBS synthesis and characterization

In the first step, 10 mg stearic acid (140 mM), 1.25 mg N-hydroxysuccinimide (NHS - 43 mM), 1 mg 1-ethyl-3-(3-dimethylaminopropyl)carbodiimide (EDC - 26 mM) were dissolved in 0.25 mL dimethylformammide (DMF) anhydrous and kept reacting overnight at 45°C. The reaction mixture was diluted with 1 mL chloroform and washed with 4 mL 0.1 M HCl to eliminate water soluble compounds. The organic phase was treated with $MgSO_4$ and dried under nitrogen steam and checked through TLC (0.5% triethylamine in chloroform): being in excess of stearic acid, it was a mixture of the reagent and of activated stearic acid (36%). In a second step, 6.1 mg of first step reaction product (corresponding to 2.2 mg activated stearic acid - 12 mM), 2 mg MBS (12 mM), 12 mg diamino-PEG (12 mM), 12 µL triethylamine (237 mM) were dissolved in 0.5 mL chloroform and kept reacting overnight at 45°C. The reaction mixture was dried under nitrogen steam; the residue was dissolved in 400 µL ethanol, and 1.6 mL 1 M HCl was added to precipitate un-reacted stearic acid from the first step reaction. The obtained solution underwent size exclusion through a Sephadex G10 column to eliminate compounds with Mw<500. ST-PEG-MBS was purified from un-reacted diamino-PEG through Dowex 50WX4 resin. The purified solution was freeze dried.

The obtained conjugate (6.4 mg) was checked through TLC (acetic acid/chloroform/methanol 1/84/15) and characterized by ^{1}H-NMR and HPLC. 300 MHz ^{1}H spectra were recorded on a Bruker (Billerica, MA, USA) 300 Avance instrument at 25 °C. HPLC analyses were performed with a YL9100 HPLC system equipped with a YL9110 quaternary pump, a YL9101 vacuum degasser and a YL9160 PDA detector, linked to YL-Clarity software for data analysis (Young Lin, Hogye-dong, Anyang, Korea), and, alternatively, with a LC10 HPLC UV system (Shimadzu, Tokyo, Japan) equipped with a evaporative light scattering (ELSD) detector (Sedex 10, Sedere, Altforville, France), linked to a Class LC10 software for data analysis. Column was a Teknocroma (Barcelona, Spain) C18 mediterranea Sea 25 x 0.46 cm, PDA wavelengths were set at 220 - 290 nm, eluent flow was 1 ml/min, gradient was 0 min: 100% water, 20 min: 100% acetonitrile, 30 min: 100% acetonitrile, 35 min: 100% water.

TLC (0.5% triethylamine in chloroform): stearic acid R_f 0.2, NHS activated stearic acid R_f 0.46. TLC (acetic acid/chloroform/methanol 1/84/15): ST-PEG-MBS Rf 0.29, stearic acid Rf 0.9, MBS Rf 0.9, diamino-PEG Rf 0.02.

HPLC-PDA. ST-PEG-MBS R_t=14.6 min. HPLC-ELSD ST-PEG-MBS R_t=15.0.

^{1}H NMR. The maleimide protons were detected at 6.5 ppm, and the stearic moiety could be easily evidenced at high field (1.2 ppm multiplet for the alkylic chain and 0.9 ppm for the methyl moiety). Both maleimide and stearic moiety were linked to diamino-PEG in 1:1 molar ratio.

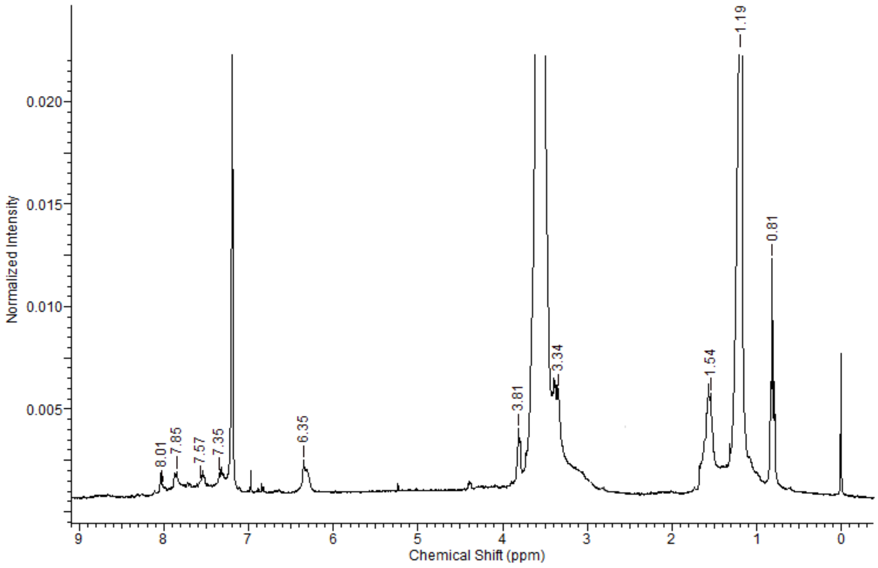

Figure S1. ^{1}H-NMR spectrum of ST-PEG-MBS

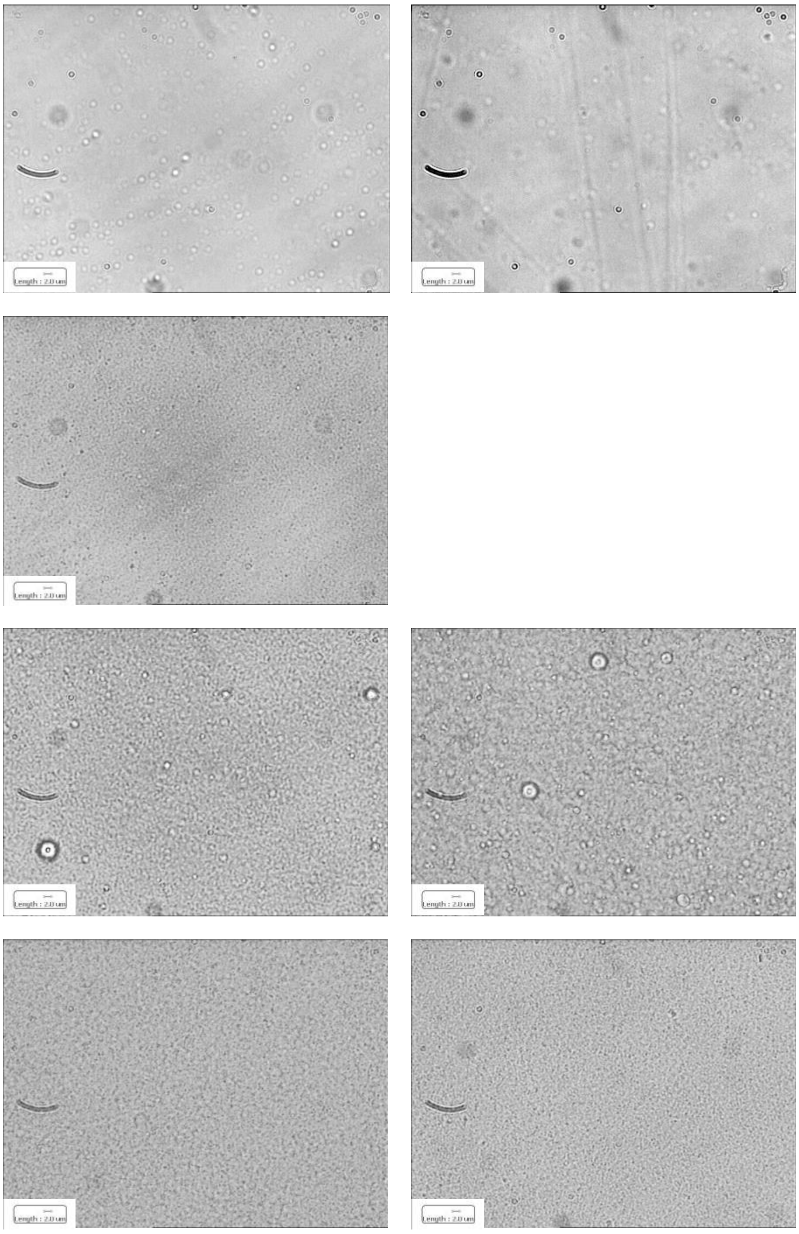

CMF

Blank IL

CMF wrapped IL before extrusion

CMF wrapped IL after extrusion

B16-F10 D4M

Figure S2. Optical microscopy of B16-F10 and D4M cell membrane fragments (CMF) wrapping of Intralipid® 10% (IL). 630X magnification. Scale bar: 2 μm

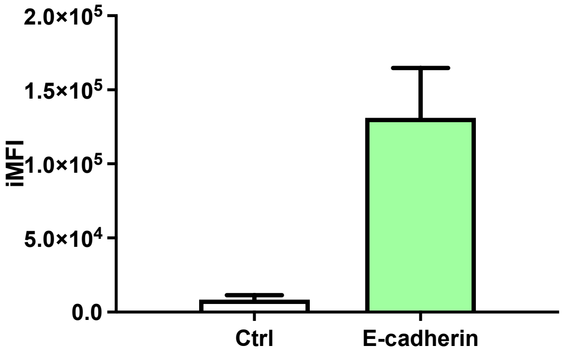

Figure S3. Flow cytometry of B16-F10 cells stained by immunofluorescence with E-cadherin antibody

Western blot densitometry

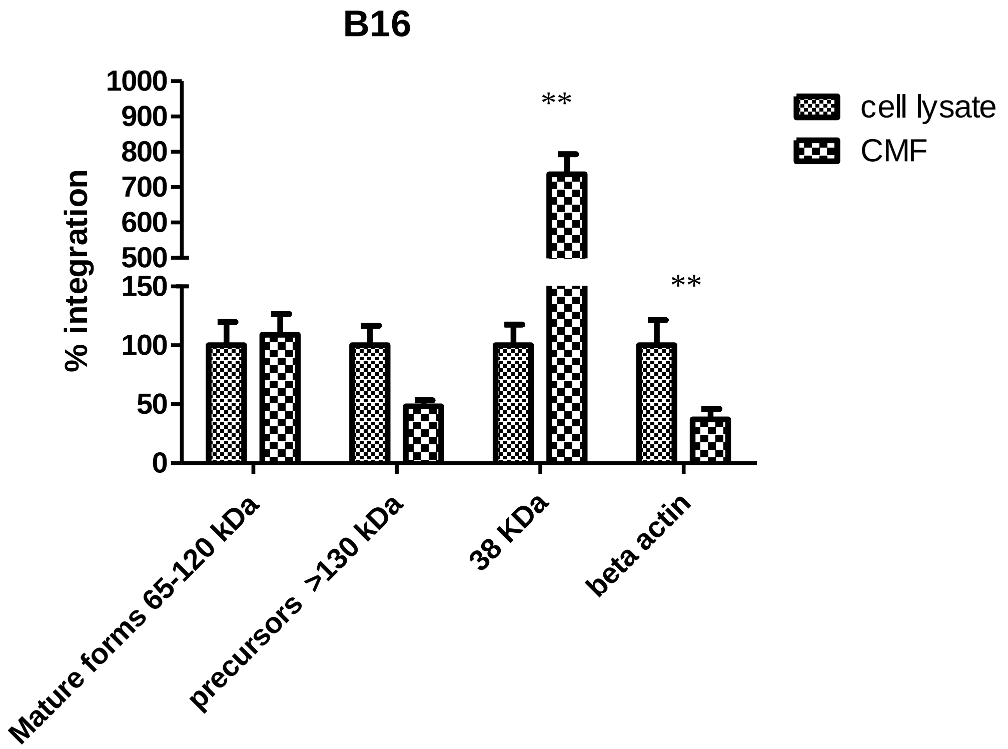

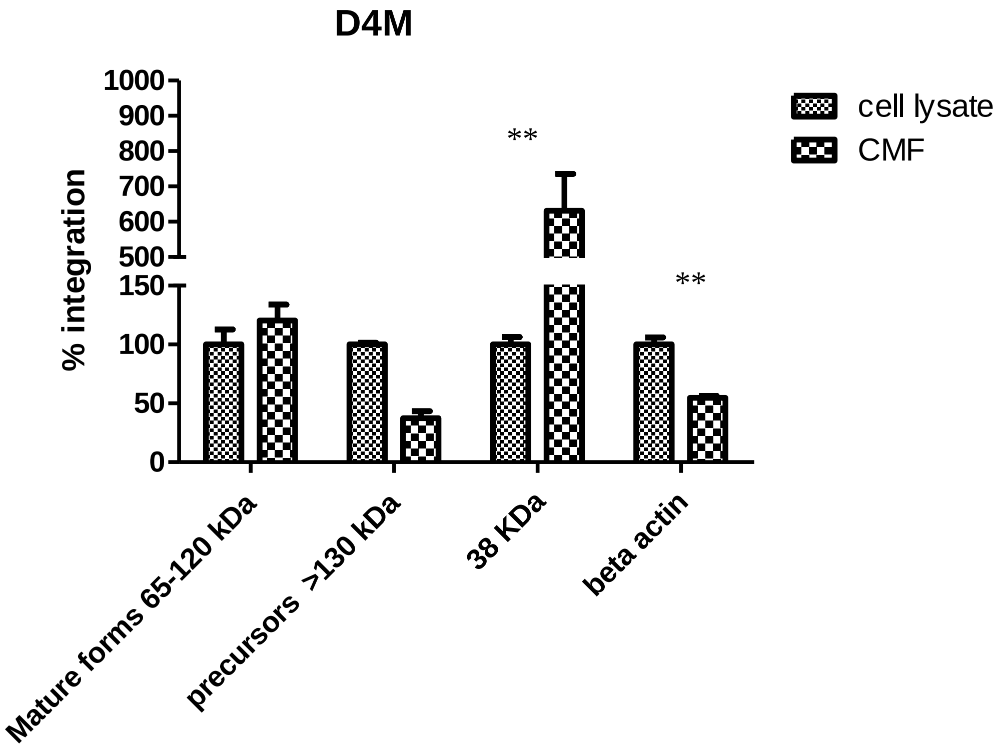

**

Figure S4. Densitometric analysis of B16-F10/D4M cell lysate and the corresponding CMF WB showed in Fig 3. We quantified the mature forms of cadherins (60-120 kDa), the cadherin precursors (>130 kDa), the ~ 38 kDa size band, a possible plasma membrane embedded fragment of E-cadherin, and the cytosolic marker β-actin. Data were normalized with the densitometry measurement mad over the entire line between 5 and 245 kDa. Values are indicated in percentage of control values as the mean ± SD of three independent experiments. ** $p \leq 0.01$ vs. C.

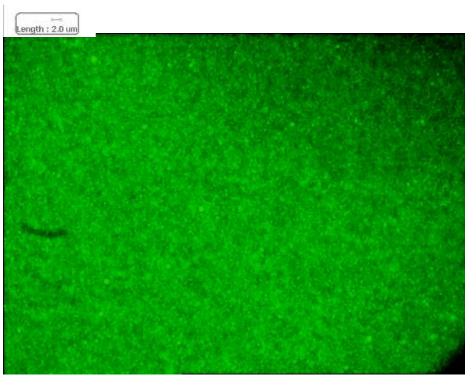

Figure S5. Fluorescence microscopy of 6-CUM fluorescently labelled Intralipid® 10% (IL). 630X magnification

Optimization of ration between B16-F10 cells and IL

With the final aim to define the optimal cell/IL ratio to be used in IL functionalized internalization studies, a preliminary experiment was conducted with probe-labelled blank IL to 6-CUM. In the study considered, B16-F10 cells were incubated for 1 h in an incubator at 37°C with 5%CO_2 with the following different conditions: 1) 1 μL IL *vs* 1 x 10^5 cells 2) 1 μL IL *vs* 5 x 10^5 cells 3) 1 μL IL *vs* 1 x 10^6 cells 4) 1 μL IL *vs* 5 x 10^6 cells 5) 1 μL IL *vs* 1 x 10^7 cells.

At the end of incubation, all the conditions were washed with PBS and analysed with an Accuri C6 (BD Biosciences, Milan, Italy) flow cytometer (considering 10,000 events and medium flow rate). Any cell debris with low forward light scatter (FSC) and side light scatter (SSC) were excluded from the analyses. Data are expressed as fluorescence of 6-CUM over the cell number (Fig s5a) and as mean integrated fluorescence intensity iMFI (Fig s5b).

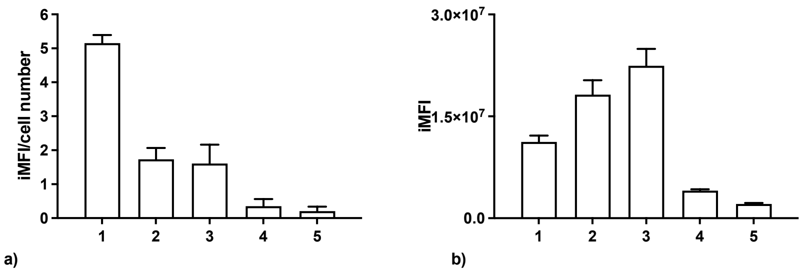

Figure S6. Flow cytometry evaluation of internalization of fluorescently labelled Intralipid® 10% (IL) in B16-F10 cells with different condition set: a) fluorescence of 6-CUM over cell number; b) mean integrated fluorescence intensity (iMFI). Conditions: 1) 1 μL IL *vs* 1 x 10^5 cells 2) 1 μL IL *vs* 5 x 10^5 cells 3) 1 μL IL *vs* 1 x 10^6 cells 4) 1 μL IL *vs* 5 x 10^6 cells 5) 1 μL IL *vs* 1 x 10^7 cells
